# Engaging research ethics committees to develop an ethics and governance framework for best practices in genomic research and biobanking in Africa: the H3Africa model

**DOI:** 10.1186/s12910-019-0398-2

**Published:** 2019-10-18

**Authors:** Paulina Tindana, Aminu Yakubu, Ciara Staunton, Alice Matimba, Katherine Littler, Ebony Madden, Nchangwi Syntia Munung, Jantina de Vries, as members of the H3Africa Consortium

**Affiliations:** 1 Department of Health Policy, Planning and Management, School of Public Health, College of Health Sciences, University of Ghana, Legon, Ghana; 2 Navrongo Health Research Centre, Ghana Health Service, Navrongo, Ghana; 3 Department of Health Planning, Research & Statistics, Federal Ministry of Health, Abuja, Nigeria; 4 School of Law, Middlesex University, Hendon, The Burroughs, London, UK; 5 Advanced Courses and Scientific Conferences, Wellcome Genome Campus, Hinxton, UK; 6 Global Policy, Wellcome Trust, London, UK; 7 Division of Genomic Medicine, National Human Genome Research Institute, National Institutes of Health, Rockville, MD USA; 8 Department of Medicine, University of Cape Town, Observatory, Cape Town, 7925 South Africa

## Abstract

In the past decade, there has been an increase in genomic research and biobanking activities in Africa. Research initiatives such as the Human Heredity and Health in Africa (H3Africa) Consortium are contributing to the development of scientific capacity and infrastructure to support these studies on the continent. Despite this growth, genomic research and biobanking have raised important ethical challenges for key research stakeholders, including members of research ethics committees. One of these is the limited ethical and regulatory frameworks to guide the review and conduct of genomic studies, particularly in Africa. This paper is a reflection on a series of consultative activities with research ethics committees in Africa which informed the development of an ethics and governance framework for best practices in genomic research and biobanking in Africa. The paper highlights the engagement process and the lessoned learned.

## Background

The Human Heredity and Health in Africa (H3Africa) Initiative [1] is a pan-African research initiative which aims to contribute to the development of scientific capacity and infrastructure to support the conduct of genomic research and biobanking on the African continent. Some of the key features of this initiative include its collaborative nature, bringing together interdisciplinary teams of researchers, institutions and communities from across the continent, the collection of human biological specimens including blood for DNA extraction, and the broad sharing of samples and data for secondary research use. Genomic research and biobanking raise important ethical questions such as those related to: what should count as valid consent for data and sample sharing; how to maintain participants’ privacy; how to ensure that the voice of the population in which the study is being done is heard through community engagement; ownership of samples and data; and benefit sharing [2–5]. Although many of these issues are not unique to the African context, a key ethical challenge in the conduct of genomic research and biobanking in Africa is the limited ethics and regulatory guidance to inform the design, review and conduct of these studies, on the continent and internationally. In the first round of funding for H3Africa (2013–2017), it emerged that many African countries lacked the appropriate frameworks to support these studies and many research ethics committees (RECs) were not familiar with reviewing proposals for genomic research and biobanking [6–8]. For example, the concept of broad consent, which allowed potential research participants to give consent for the broad use of their samples and data including secondary use and was the proposed model for H3Africa projects, became a subject of intense debate among key research stakeholders including RECs. Questions were raised about its appropriateness in the African research context since RECs will not be able to track the status of stored samples when they leave the Continent. Also, there were concerns that given the level of literacy in Africa, participants would not understand the rationale for future use of samples and data. These concerns were similar to those reported elsewhere [7, 9].

The H3Africa Consortium adopted several approaches to address these ethical challenges through the work of the H3Africa Ethics and Regulatory Issues Working Group and the H3Africa Community Engagement Working Group (CEWG), of which the authors of this manuscript are a part. These Working Groups are responsible for developing appropriate ethics and community engagement policies and guidelines [10, 11] for H3Africa projects as well as supporting empirical studies addressing the ethical, legal, social and cultural implications of genomic research and biobanking in Africa. These two groups have since been joined and in the remainder of this paper, we will refer to these groups as ‘the Working Group’.

Given the limited ethics guidance available to support these studies, particularly on the African continent and repeated calls for guidance from ethics committees and other stakeholders, the Working Group deemed it necessary to develop a framework to address the growing concerns about the conduct of genomic studies and biobanking in Africa [6]. The purpose of the ethics framework was to provide key elements of best practice to guide the design, review and conduct of genomic research and biobanking in Africa and to serve as a guide for the development of specific institutional and national guidelines and policies for genomic research and biobanking across the continent [12]. But when considering the development of such a framework, what became obvious is that central to its development should be the voices and perspectives of different stakeholders that would work with and apply it. We thought this was important not only to increase the relevance and legitimacy of the framework, but also because broad engagement is a key element of ensuring that research fulfils the goals of justice [13]. Thus, in the process of developing this document we engaged members of RECs, as stewards in promoting ethical research, researchers and other key African stakeholders including national and regional regulatory authorities.

In this paper we describe this engagement process with REC members and the other key stakeholders as well as lessons learned in the development of an ethics and governance framework for best practice in genomic research and biobanking in Africa. A separately published manuscript discusses the main content of the framework and is open access [14].

## Discussion

### Our engagement approach: ethics consultation and deliberative workshops

Community and stakeholder engagement is increasingly gaining prominence as an important process that can support the successful implementation of genomic research and biobanking, particularly in facilitating the sharing of information with target communities and stakeholders and addressing the ethical issues that arise in practice [15–18]. One key approach to community and stakeholder engagement is consultation, which provides two-way communication and a platform for stakeholders to deliberate on key issues of common interest. While project-specific community engagement activities with members of the lay public were being conducted across the H3Africa Consortium [19, 20], the need to engage with RECs became increasingly obvious for two reasons. First, genomic research is collaborative, and RECs have a gatekeeper role to ensure the safety of research participants. With this, they have the power to delay or otherwise obstruct research or to approve and facilitate research. It is now well-established that many of the ethics committees on the continent are deeply uncomfortable with the export, storage and re-use of samples and data, not in the least out of fear that these practices would fuel exploitation of African researchers and populations [21]. For H3Africa projects seeking ethics approval, these fears translated into long delays in obtaining ethics approval. For the Working Group, one way of addressing these fears was to engage with members of RECs to understand better the root of these fears and to work with them in finding constructive solutions. Second, RECs are the most visible entity protecting the values of the institutions and communities involved in research. Although there are obvious limitations to their ability to act in this capacity, the Working Group felt that it was crucially important that the views of these committees were heard and taken seriously in the design of guidance developed by the H3Africa Consortium.

The Consortium’s approach to engaging with the key stakeholders was to organise a series of consultation workshops to identify the key practical and ethical challenges from the perspective of those involved in reviewing and regulating these studies and to solicit their views on how these challenges can be addressed. The format for these workshops included presentations, plenary and group discussions. To support a more deliberative process, the workshops were structured in a way that allowed more time for both open and group discussions. The format also involved presenting various views on the subject matter and eliciting the views of workshop participants on what would count as best practices. In what follows, we describe the components of these consultation workshops, lessons learned and how they contributed to the development of the ethics and governance framework for best practice in genomic research and biobanking in Africa.

### 1st consultation meeting: Cape Town South Africa, June 2014

The first consultation workshop was held in Cape Town, South Africa in June 2014. Sixty members from 40 RECs in 18 African countries, principal investigators and funders of the H3Africa Consortium attended the workshop. REC members were purposively selected from RECs that were involved in the review of H3Africa research protocols. The response to the workshop invitations were generally positive. Only a few invitees declined to attend the workshops due to conflicts with the proposed dates.

The focus of this workshop was to provide a platform for members to share their experiences in the review of H3Africa protocols, including the concerns they have with some of the key features of the projects such as sample and data sharing and the use of broad consent. Two brief presentations were made at the beginning of the workshop. The first focused on the scientific rationale for conducting genomic research in Africa, an overview of the H3Africa projects, the governance structures that have been established by the Consortium on consent, community engagement and data sharing. The second presentation focused on the key ethical issues highlighted in the literature and the various perspectives on consent for genomic research globally. It also provided an opportunity for participants to learn from research practices outside the H3Africa Consortium and to deliberate on the best way forward in addressing the key ethical issues arising in practice.

The key issues that dominated the discussions were the concept of broad consent [22], community engagement and the role of local RECs in decisions on secondary use of samples. It became apparent that the lack of clarity on the definition of broad consent and how to differentiate it from blanket consent, which has no restriction on future secondary use, was creating anxieties among the REC members. The workshop provided an opportunity to explore the nature of these concerns, get a sense of ethics committee’s general perceptions on genomic research and biobanking and identify potential pitfalls that would need to specifically be addressed in H3Africa guidelines and policies. Importantly, the meeting also offered an opportunity for the H3Africa Consortium to receive input on its draft data and sample sharing policy.

The outcome of this workshop informed the revision of the H3Africa Informed Consent Guidelines [10], which clarified the concept of broad consent. Two empirical ethics projects focusing on community engagement [23] and an analysis of the consent forms used across the H3Africa Consortium [24] were also conducted. Furthermore, the meeting led to a revision of the draft H3Africa sample and data sharing policy, particularly with regards to including a process for ensuring regular feedback to the ethics committees that first approved sample collection about data and sample use requests.

### 2nd consultation meeting: Livingstone, Zambia, may 2015

Following the success of the first consultation workshop and the need for further discussions on what would constitute best practices for conducting genomic research and biobanking in Africa, a second workshop was held in Livingstone, Zambia in May 2015 [24]. The nature of discussions about the consent processes for African genomic research at the first meeting led us to focus this second meeting more explicitly on broad consent. Specifically, this meeting sought to explore perspectives on the use of broad consent but also the nature of the governance framework that should accompany it. To ensure some continuity of the engagement process, participants of the first consultation meeting were invited to this meeting. The need to start including regulatory bodies was seen as necessary to ensuring that the future governance framework would inform the development of national frameworks across Africa. This was partly a recommendation made by participants in the first Consultation meeting.

At this second meeting, there were clear indications that the perspectives of members of RECs on broad consent as the best option for genomic research and biobanking had evolved since the first meeting, with greater apparent support for this type of consent [24]. The discussions had moved from ‘what are the ethical issues we are concerned about’ to ‘how should we address the ethical issues arising in practice’ and ‘what governance mechanisms should support the use of broad consent’. The meeting called for clear guidelines and regulations across the African continent. The meeting also called for harmonisation of approaches and guidelines across the continent. The reason for this is that much of the research conducted in Africa happens in collaboration between African partners or with non-African partners. In those cases, significant differences in approaches between partners has been known to cause delays and obstruct collaboration. There is also a concern that if only some countries adopt strict regulations (for instance, stipulating that all collaborative research requires a partner for the country) that international partners would avoid those countries and rather work with researchers in countries where regulation is less strict. The discussions at this workshop informed a project by the Working Group to review existing guidelines and regulations on genomic research and biobanking across Africa [6]. Overall, the meeting concluded with an affirmation of our view that the absence of guidance documents that are relevant to the African context to help ethics committees make sense of genomic research and biobanking research proposals, formed a real obstruction to committees’ ability to adequately review such proposals. The consultation process provided a great opportunity to clarify some of the disagreements on the definitions of key terms such as broad consent for data sharing, which had generated considerable debate. The issues raised by workshop participants formed the basis of the key principles and elements outlined in the framework document.

### Developing the framework

Taking the call for guidance for African genomic and biobanking research seriously, we agreed that whilst it was important to support such a process, we equally did not feel that the expertise of the relatively small number of active members of the Working Group had sufficient experience to develop such a document alone. For this reason, we decided to build on the relations developed during the H3Africa consultation meetings and involve key ethics committee members in the drafting process. After preparing an initial draft in the group, we sourced external funding that allowed us to organise a two-day workshop in March 2016. This meeting involved members of ethics committees in three countries (Botswana, Ethiopia and Uganda), representatives of the two funding bodies (NIH and the Wellcome Trust), a member of the South African Academy of Science, an African philosopher, and selected members of the Working Group. During this meeting, we fostered discussion on all components of the framework, including critical questions of definition, scope and philosophical orientation.

The team deliberated on the foundational philosophy of the document, the principles that would serve as the basis of the governance mechanism that was idealised and the elements that explained the operationalization of such principles in practice. To avoid reinventing the wheel, the team drew on existing ethics frameworks such as the Framework for the Responsible Sharing of Genomic and Health-Related Data developed by the Global Alliance for Genomics and Health (GA4GH) [25] as well as the OECD Guidelines on Human Biobanks and Genetic Research Databases [26], the Wellcome Trust Framework on the Feedback of Health-Related Findings in Research [27], the European Commissions’ reports on Global Governance of Science [28], the Ethical and Regulatory Challenges to Science and Research Policy at the Global Level [29] and country- specific ethics guidelines from 23 African countries, some of which were specific to genomic research and biobanking [6].

We used the resulting draft to engage broadly with stakeholders involved in genomic research and biobanking internationally and in Africa. We engaged quite extensively, for instance with the Bridging Biobanking and Biomedical Research across Europe and Africa (B3Africa) [30] and TRUST projects funded by the European Union, which focus on biobanking in Africa and on international research ethics respectively. We also received input from the P3G Consortium [31], which develops policy tools and provides ethics support for public and private health research initiatives. Inputs were integrated into the draft Framework.

### 3rd consultation meeting: Dakar, Senegal, may 2016: consensus building on ethics framework

The 3rd Consultation meeting was convened in Dakar, Senegal in May 2016 to deliberate on the draft ‘Ethics and Governance Framework for Best Practice in Genomic Research and Biobanking in Africa’ as part of the process of finalising the revised version of the Framework and to identify the mechanism to support its implementation across the continent.

In all, 40 participants from Ministries of Health, National Ethics Councils, REC members, research institutes, the African Academy of Sciences, the National Institutes of Health and the Wellcome Trust participated in this workshop. Participants discussed each of the elements in the draft Ethics and Governance Framework in groups and provided feedback on what needed to be changed or improved and what was missing. One of the key areas of discussion related to the African philosophical orientation that should underpin the key elements proposed in the framework. The draft document had relied on the Ubuntu philosophy [32], which highlights the interrelatedness and interconnectedness of people, as the basis for what should count as good ethical practice in Africa, particularly on the need to share samples and data. However, some participants at the workshop felt that Ubuntu is limited to South African philosophy and that it has strong political connotation in that country. They suggested that it might be necessary to identify a common African philosophical thought that cultures across the continent could identify with. Based on these discussions, Ubuntu was removed from the final document and replaced with text highlighting communal and solidarity-based worldviews, which are common across many cultures in Africa.

At the end of this third consultation meeting, a communiqué was developed based on the key recommendations from the stakeholders’ discussions. The document called for greater involvement and engagement with regional governance organisations such as the African Union and the African Academy of Science to push for African governments to support funding on genomic research and biobanking and to facilitate the development of relevant national and regional guidelines and policies to support genomic research and biobanking across the continent.

### Other matters arising and next steps

The final Ethics and Governance framework document has been shared with all the participants who attended the three workshops as well as the wider research community online. [12] As part of its dissemination efforts, members of the Working Group have presented the framework at several national and international conferences, including the 10th International Association of Bioethics Congress held in Edinburgh in June 2016. An implementation guide is being developed to support the uptake of this framework across the continent.

### Lessons learned

Our experience has taught us several valuable lessons that can guide future engagement activities with RECs, regulators and policy makers on the African continent in line with our approach. First of these is the importance of funding and support in organising these events. Funding for these workshops was not available in any one H3Africa project. Rather, the funding bodies backing H3Africa (the NIH and the Wellcome Trust) supported these initiatives with funding they sourced purposively for these meetings. We would recommend that this type of funding support should be a feature of research initiatives and should consider engagement with RECs as an important step to addressing the key ethical challenges that would arise in practice. Second, the Working Group received support from many different stakeholders responsible for its success – researchers, the H3Africa steering committee and external advisory board, and the H3Africa funders. Without such support, organising these meetings and ensuring that lessons learnt were translated into the guidance developed, would have been difficult if not impossible.

Another lesson is the importance of having ‘champions’ that believe in the value of engagement and, equally important, that believe in the importance of medical research to help address illnesses affecting African populations. Over the course of our work, we identified two kinds of champions – those involved in H3Africa research and the Working Group and members of research ethics committees outside it. In terms of the first, there was a small but significant group of enthusiastic individuals, each with their own day-job, who believed in the value of engagement and who were willing to put in the hours to make it happen. But more importantly, through the course of our Consultation Meetings, we identified members of ethics committees that were experienced with genomic research and biobanking who spoke out and added significant value to the meeting deliberations and who were keen to actively participate in the drafting of the Framework for instance. Importantly, they were responsive to email and willing to travel. These ‘champions’ became our primary source of engagement with the community of ethics committees that we were seeking to engage. Without their perspectives, the development of the Framework would not have been nearly as successful.

Early engagement with policy makers and regulatory bodies is also very important to ensure mutual understanding of the key concerns these stakeholders might have and to ensure that policies are developed appropriately to address these issues.

As these workshops were more consultative, the issues raised by the participants informed the key principles and elements outlined in the framework document. We believe that these processes served several purposes; by strengthening understanding of genomic research and the key ethical considerations that should guide the design, review and implementation of these projects. The workshops also demonstrated to the RECs that the H3Africa consortium was not only focusing on the scientific aspects of the project but also concerned about promoting the ethical conduct of these projects by identifying and addressing them as they arise through appropriate governance structures.

Not all RECs in Africa were involved in these consultation workshops and while concerns about the appropriateness of broad consent in Africa evolved overtime, we acknowledge that the debates on broad consent are not over. There is therefore the need to sustain these consultation workshops with RECs and regulatory bodies and undertake facilitation in a way that allows diverse and dissenting voices to be captured. These processes could contribute to building trust between these various research actors and facilitate the ethical conduct of genomic research in Africa. It will also be important to conduct further empirical studies to continue to build the evidence-base to support consent models in Africa.

The framework has received some publicity in a Nature editorial [33], and in the Journal of America Medical Association (JAMA) [34] amongst others. More anecdotally, in empirical research we conducted with REC members in Ghana, Uganda [35] and Zambia [36] we heard the H3Africa engagement process and the framework referred to as a source of knowledge and as instrumental in the development of appropriate review processes. Going forward, the Working Group plans to monitor the impact of this framework through a continuous process of engagement with members of ethics committees and national policy makers.

## Conclusion

This paper has demonstrated a bottom-up approach to developing an ethics and governance framework and the role of stakeholder engagement in facilitating the development process. Using a consultation approach to engage REC members and regulatory authorities ensured that the concerns of these key stakeholders were considered and used to inform the development and review of H3Africa policy documents and guidelines. It has also highlighted the need to create more opportunities for communication between researchers, RECs and regulatory bodies across the continent to facilitate the ethical conduct of research. Going forward, the H3Africa Consortium is keen to support the implementation phase of the framework. Given the limited opportunities for RECs to meet and network on the continent, these consultation workshops have set the stage to sustain face to face interactions. We call on the African Union and the African Academy of Sciences to support an initiative that will support a strong network of RECs across Africa. This should inform fostering a harmonised approach to the regulation of genomic research and biobanking on the continent and the development of ethics review equivalency agreements between committees.

## Data Availability

Not Applicable.

## Abbreviations

CE: Community Engagement
H3Africa: Human Heredity and Health in Africa
REC: Research Ethics Committees
